# Mining and validation of novel simple sequence repeat (SSR) markers derived from coconut (*Cocos nucifera* L.) genome assembly

**DOI:** 10.1186/s43141-022-00354-z

**Published:** 2022-05-16

**Authors:** Reina Esther S. Caro, Jesmar Cagayan, Roanne R. Gardoce, Anand Noel C. Manohar, Alma O. Canama-Salinas, Ramon L. Rivera, Darlon V. Lantican, Hayde F. Galvez, Consorcia E. Reaño

**Affiliations:** 1 Institute of Plant Breeding (IPB), College of Agriculture and Food Science, University of the Philippines Los Baños, College, 4031 Los Baños, Laguna Philippines; 2 Philippine Genome Center — Program For Agriculture, Livestock, Fisheries and Forestry, University of the Philippines Los Baños, College, Los Baños, 4031 Laguna Philippines; 3 Philippine Coconut Authority — Zamboanga Research Center, San Ramon, 7000 Zamboanga City, Philippines; 4 Institute of Crop Science, College of Agriculture and Food Science, University of the Philippines Los Baños, College, 4031 Los Baños, Laguna Philippines

**Keywords:** Bioinformatics, Catigan green dwarf genome, Coconut (*Cocos nucifera* L.), Marker-assisted breeding, SSRs

## Abstract

**Background:**

In the past, simple sequence repeat (SSR) marker development in coconut is achieved through microsatellite probing in bacterial artificial chromosome (BAC) clones or using previously developed SSR markers from closely related genomes. These coconut SSRs are publicly available in published literatures and online databases; however, the number is quite limited. Here, we used a locally established, coconut genome-wide SSR prediction bioinformatics pipeline to generate a vast amount of coconut SSR markers.

**Results:**

A total of 7139 novel SSR markers were derived from the genome assembly of coconut ‘Catigan Green Dwarf’ (CATD). A subset of the markers, amounting to 131, were selected for synthesis based on motif filtering, contig distribution, product size exclusion, and success of in silico PCR in the CATD genome assembly. The OligoAnalyzer tool was also employed using the following desired parameters: %GC, 40–60%; minimum ΔG value for hairpin loop, −0.3 kcal/mol; minimum ΔG value for self-dimer, −0.9 kcal/mol; and minimum ΔG value for heterodimer, −0.9 kcal/mol. We have successfully synthesized, optimized, and amplified 131 novel SSR markers in coconut using ‘Catigan Green Dwarf’ (CATD), ‘Laguna Tall’ (LAGT), ‘West African Tall’ (WAT), and SYNVAR (LAGT × WAT) genotypes. Of the 131 SSR markers, 113 were polymorphic among the analyzed coconut genotypes.

**Conclusion:**

The development of novel SSR markers for coconut will serve as a valuable resource for mapping of quantitative trait loci (QTLs), assessment of genetic diversity and population structure, hybridity testing, and other marker-assisted plant breeding applications.

## Background

Coconut (*Cocos nucifera* L.) is one of the most economically important crops in the Philippines. In 2017, the country produced 14.05 million metric tons of coconut, and the value of production hits 120.3 million pesos [19]. The Philippines remained to be the top global supplier of coconut copra and desiccated coconut in both volume and total USD value as of 2010 [4]. Coconut oil, one of the many diversified products of coconut, ranked first among the top ten agricultural exports of the Philippines comprising 21.9% of the total agricultural exports in 2015 [18].

Coconut is situated across the tropical and subtropical latitudes that are accessible to the equatorial Pacific Ocean current which possibly favored the evolution and dispersal of coconut. Coconut palms thrive well in humid coastal environments at about 18° of latitude north or south of the equator where there is fertile soil, favorable temperature, and year-round rainfall [5]. Coconut belongs to the Indian center (II) and Indo-Malayan subcenter (II-A, where the Philippines belongs) in Vavilov’s center of origin of cultivated plants [26]. It is generally classified into two types: tall and dwarf. The tall types are generally allogamous (heterozygous) or cross-pollinating, slow to mature; flower at 6–10 years after planting, and with an economic life of 60–70 years. Dwarf types, on the other hand, are highly autogamous (homozygous), or mainly self-pollinating, early to flower at around 4–6 years after planting with a productive life of 30–40 years [2, 6, 12].

Coconut is a diploid with 32 chromosomes (2*n* = 2× = 32). It belongs to the family Arecaceae (Palmaceae) in the subfamily Cocoideae and is the lone species of genus *Cocos* [17]. The estimated genome size of coconut is approximately 2.6 Gbp comprising of 50–70% repetitive sequences . Lantican et al. [9] reported the estimated genome size of ‘CATD’ to be 2.14 Gbp. The abundance of repeat contents in the coconut genome becomes advantageous in the assessment and characterization of coconut varieties/populations using molecular marker techniques. The use of molecular tools offers a more accurate assessment than the conventional way of characterizing coconut which is through morphological and agronomical traits that are mostly influenced by many environmental factors [15].

Molecular markers have established its importance as a modern breeding tool for crop improvement [7, 24, 31]. The use of molecular tools can significantly accelerate the overall duration of breeding programs for coconut improvement. One of the extensively used markers in molecular breeding and genetic diversity analyses is the simple sequence repeats (SSR). SSRs are short tandem repeats that have repeating units of di-, tri-, tetra- and pentanucleotides [20]. They are approximately 1–8-bp long, abundant, and well distributed throughout the genome on which repeat units can vary between genotypes/individuals which make it a very useful tool in fingerprinting, genotyping, and genetic diversity analyses [23].

In the past, SSR marker development in coconut was achieved through microsatellite probing in bacterial artificial chromosome (BAC) clones or using previously developed SSR markers from closely related genomes [15, 21]. These coconut SSR markers are publicly available; however, the number and distribution across chromosomes are quite limited for quantitative trait loci (QTL) mapping and genetic diversity studies. Fortunately, with the current advancements in next-generation sequencing (NGS) technologies, it has now become possible to mine SSRs across the entire genome. By using genome-wide bioinformatics prediction, we can generate a vast amount of SSR markers efficiently.

This study aims to provide a valuable resource of SSR markers for potential use in marker-assisted selection breeding for coconut.

## Methods

### Plant materials and leaf collections

Leaf samples of the coconut parental genotypes ‘Catigan Green Dwarf’ (CATD), ‘Laguna Tall’ (LAGT), and ‘West African Tall’ (WAT) and a synthetic variety denoted as SYNVAR (LAGT × WAT) used in this study were obtained from the Philippine Coconut Authority — Zamboanga Research Center (PCA–ZRC) in San Ramon, Zamboanga City, Philippines. Coconut leaflets coming from the youngest frond or the “first leaf” and are free from any pest damage were carefully chosen as samples. Three leaflets were gathered from each of the left and right portions of the midrib near the base of the frond. The samples were transported to the Genetics Laboratory at the Institute of Plant Breeding — University of the Philippines Los Baños (IPB-UPLB), Laguna, Philippines, for DNA extraction.

### Genomic DNA extraction of coconut parental genotypes

A total of eight individuals/palms of the coconut genotypes were collected (Table 1). Genomic DNA was extracted following the procedure adapted from Doyle and Doyle [3] with modifications. DNA quality and yield were determined by electrophoresis in 1% UltraPure™ agarose (Invitrogen Corp., Carlsbad, California, USA) in 1× Tris-borate EDTA (TBE) running buffer at 100 V for 40 min, 0.5 ug mL^−1^ ethidium bromide staining, and UV illumination at 300 nm using the Enduro GDS Touch Imaging System (Labnet International, Inc, Edison, New Jersey, USA). DNA concentration was estimated by visual comparison of gel fragments with known concentrations of lambda (λ) DNA molecular weight standards (Sigma-Aldrich Inc., St. Louis, Missouri, USA).

**Table 1:** Coconut genotypes used in the study for screening the SSR markers

Entry number	Coconut cultivars	Code	Palm number	Origin
1	Catigan Green Dwarf	CATD	1715	Davao City
2	West African Tall	WAT	0519	Ivory Coast
3	West African Tall	WAT	0610	Ivory Coast
4	West African Tall	WAT	0704	Ivory Coast
5	West African Tall	WAT	0720	Ivory Coast
6	Laguna Tall	LAGT	0107	Davao City
7	Laguna Tall	LAGT	0508	Davao City
8	SYNVAR (LAGT × WAT)	AN17	4017	Zamboanga City

### Development of SSR markers using the genome assembly of coconut ‘Catigan Green Dwarf’ (CATD)

**Fig. 1:**
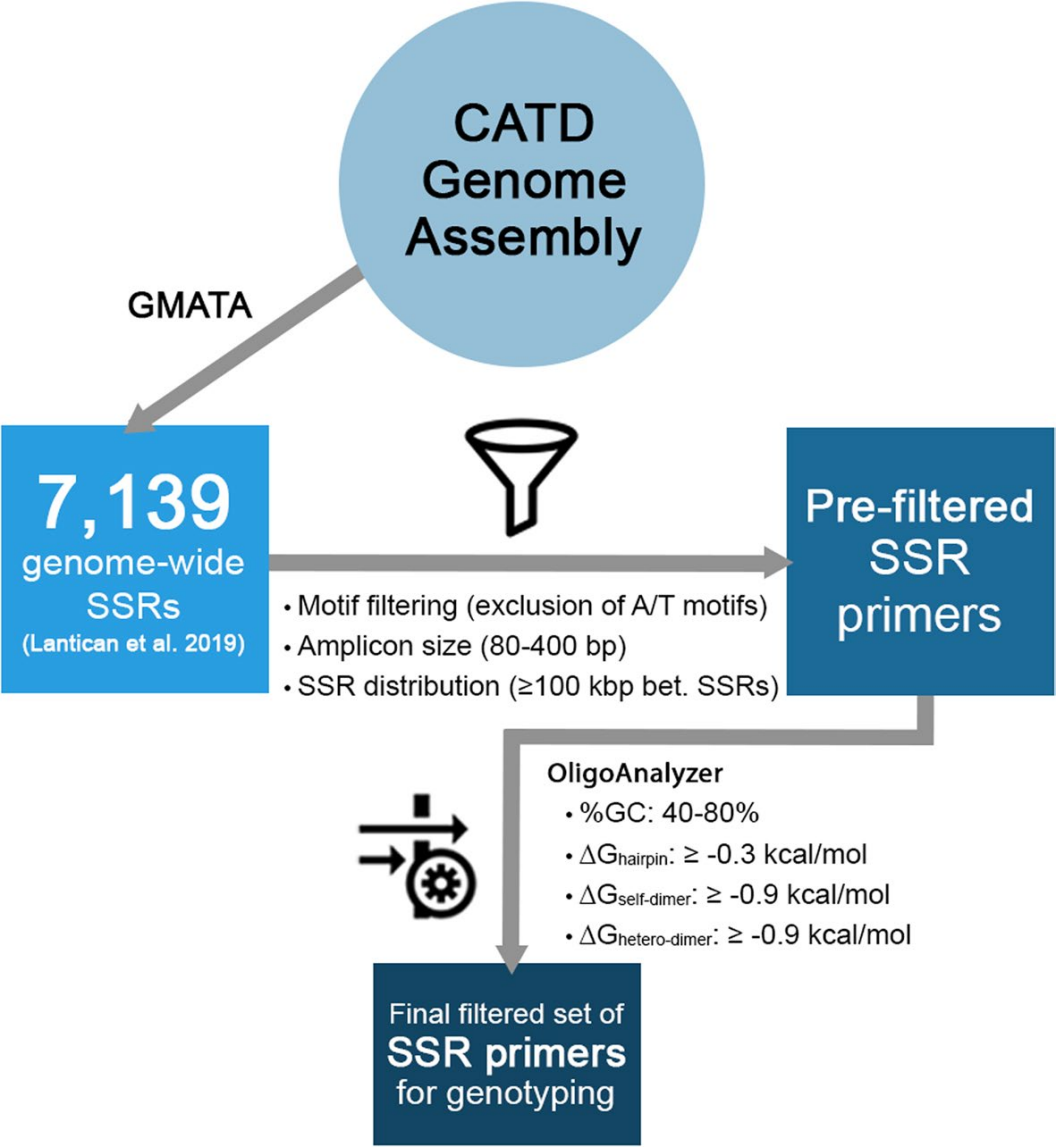
Schematic diagram depicting the SSR primer filtering pipeline

Previously, a set of 7139 novel SSRs was automatically generated based on the SSR loci annotation of the genome assembly of coconut ‘Catigan Green Dwarf’ (CATD) using GMATA software package [9, 27]. Given the vast amount of the predicted SSR markers, selection criteria were employed to obtain high-quality markers for eventual use in coconut genotyping. Motif filtering, contig distribution, and product size exclusion were used to further filter the predicted markers by manual checking. Markers with AT/AT and TA/TA repeat motifs were excluded in the selection. In silico PCR in the ‘CATD’ genome assembly [9] was then performed to ensure in vitro SSR amplification prior to synthesis [22]. OligoAnalyzer tool (Integrated DNA Technologies, Inc., Coralville, Iowa) was also employed using the following desired parameters: %GC, 40–60%; minimum ΔG value for hairpin loop, −0.3 kcal/mol; minimum ΔG value for self-dimer, −0.9 kcal/mol; and minimum ΔG value for heterodimer, −0.9 kcal/mol for further filtering of the SSRs (Fig. 1).

### PCR analysis

PCR was carried out with 10 uL reaction volume (15 ng genomic DNA, 1× PCR buffer (10 mM Tris pH 9.1 at 20 °C, 50 mM KCl, 0.01% Triton™ X-100); Vivantis Technologies, Malaysia), 1.5 mM MgCl_2_, 0.2 mM dNTPs (Promega Corporation, Madison, Wisconsin, USA), 0.2 μM forward and reverse primer (Integrated DNA Technologies Pte. Ltd., Singapore), and *Taq* DNA polymerase (Vivantis Technologies, Malaysia). The temperature profile used is as follows: initial denaturation at 95 °C for 3 min, 30 cycles of denaturation (95 °C, 30 s), annealing (45–60 °C depending on the primer pair, 45 s), extension (72 °C, 1 min), and final extension at 72 °C for 5 min. Amplifications were carried out in the Applied Biosystems Veriti™ 96-well Thermal Cycler (Thermo Fisher Scientific, Madison, Wisconsin, USA). PCR products were resolved with electrophoresis using 8% non-denaturing polyacrylamide gel in 1× Tris-borate EDTA buffer at 100 V for 60–75 min in the C.B.S. Scientific Triple Wide Mini-Vertical System™ (C.B.S. Scientific Company San Diego, California, USA) and visualized using 0.5 ug mL^−1^ ethidium bromide staining and UV illumination using the Enduro GDS Touch Imaging System (Labnet International, Inc, Edison, New Jersey, USA). Gels were scored manually for the presence or absence of bands.

## Results

**Fig. 2:**
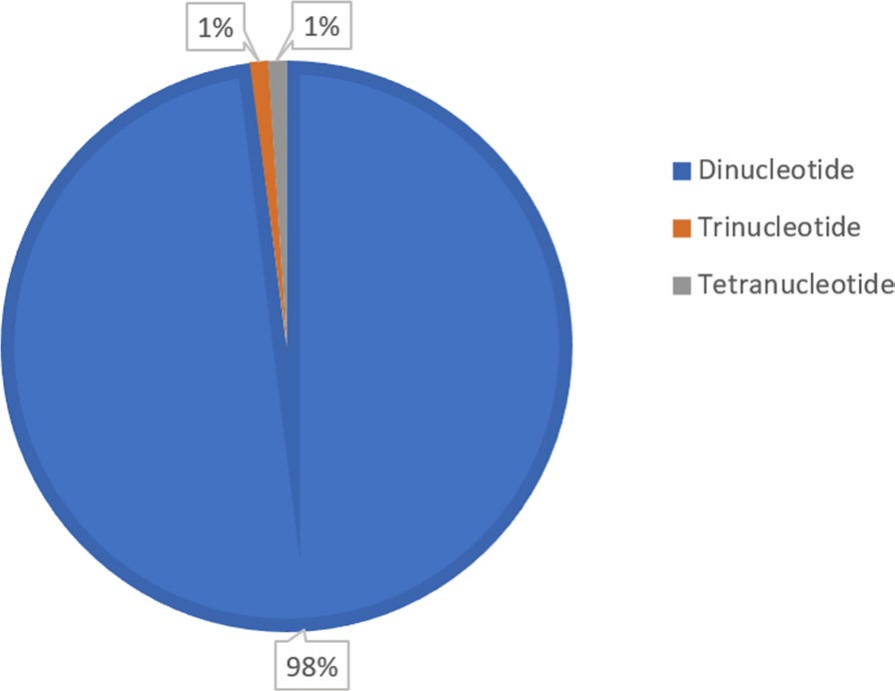
Percentage of repeat motifs of the selected SSRs

A total of 131 SSR markers were synthesized, and 98% of these were comprised by dinucleotide repeats (or 2-mer), while the remaining 2% are tri- and tetranucleotide repeats comprising of 1% each, as shown in Fig. 2. AG and GA motifs are the most abundant dinucleotide repeats found in the 131 SSR markers, with 29 and 18.3%, respectively. These are followed by CT (14.5%), TG (13.7%), TC (11.5%), AC (7.6%), and GT (3.8%) repeats. In addition, tri- and tetranucleotide repeats of AAG (1.0%) and ACAT (1.0%) were also observed.

**Fig. 3:**
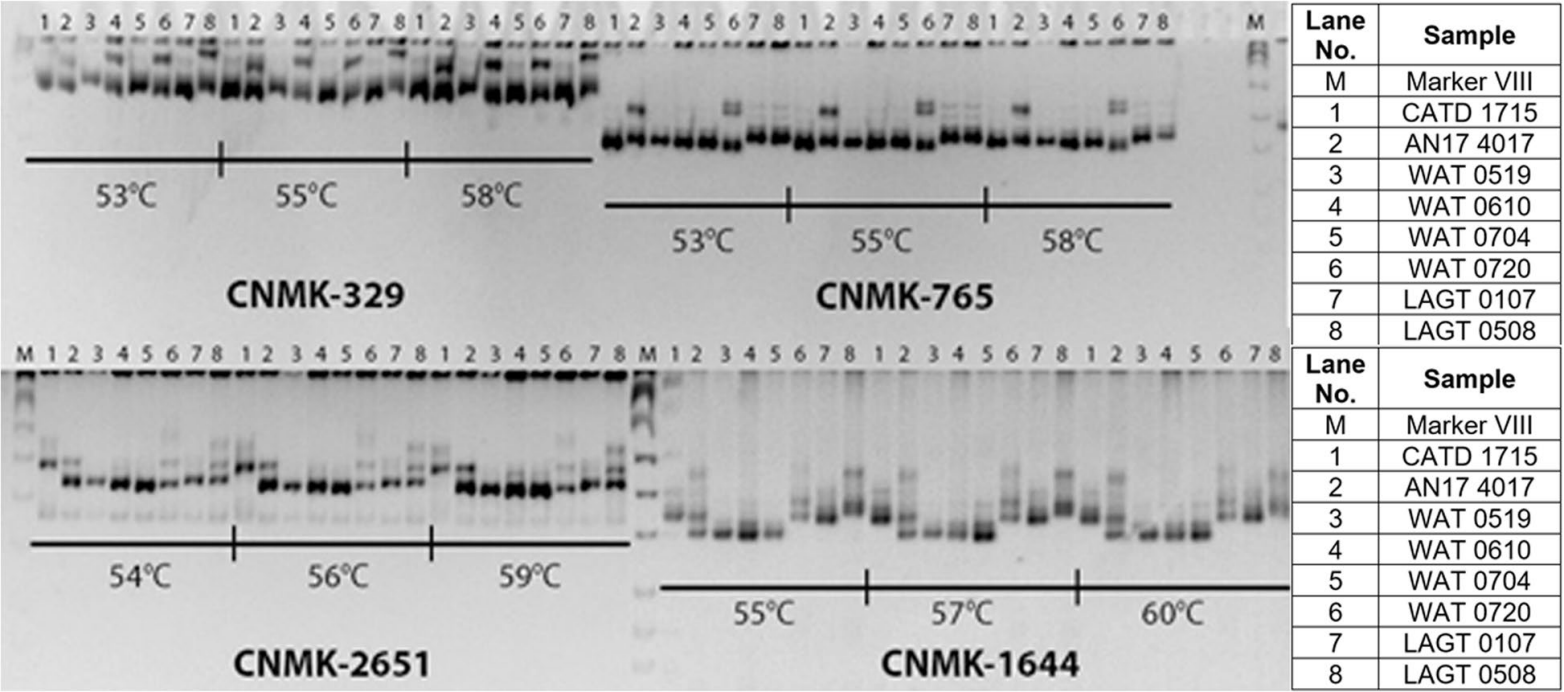
Representative gels of polymorphic SSRs optimized among coconut genotypes

**Fig. 4:**
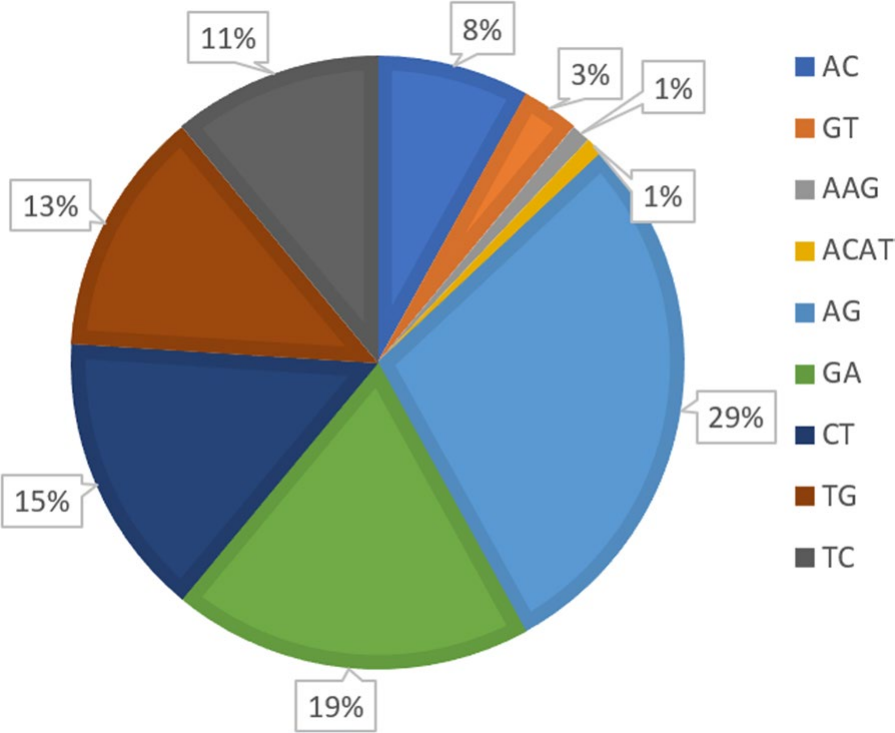
Percentage of polymorphic SSRs per motif

All SSRs showed successful amplification in coconut genomic DNA. Of the 131 SSRs, 113 (86%) were polymorphic among the test coconut varieties, while the remaining 18 (14%) were monomorphic. An average of 2.70 alleles per locus was observed across test varieties, implying a high degree of polymorphism of the selected SSRs. Representative gels of polymorphic SSRs optimized among coconut genotypes are presented in Fig. 3 on which distinct and good amplification patterns were observed. The product size of these markers ranged from 130 to 690 bp. The summary of the characteristics of the selected SSRs are presented in Table 2 which includes the name of marker, annealing temperature, repeat motif, contig distribution, product size range, and number of alleles (Fig. 4).

**Table 2:** Characteristics of the selected coconut SSRs with name, primer sequence, annealing temperature, repeat motif, contig number, and expected allele

No.	Marker ID	Primer sequence	Calculated T_**m**_ (°C)	Optimum T_**a**_ (°C)^**a**^	Expected allele size (bp)	Motif	Contig	Observed size range (bp)	Polymorphisms	No. of alleles per locus
1	CNMK7	F: GAGGGAGAGAGGCTGTGATG R: GCATGTTCAAGGCTTTGGTT	55.6	56	299	AG	0	242–260; 404–480	P	2
2	CNMK172	F: TTTCCCATCTTGATCCACCT R: TTTGATGGCTGGTGTAATGC	53.55	57	280	TG	1	280–320	P	3
3	CNMK329	F: AGTGGCTTCAAGTGGGTCAG R: CACCATCCTTGGCTTTCAAT	55.3	55	342	CT	1	320–360	P	2
4	CNMK653	F: AGCCCAAATTCGATCAACTG R: ATACATGGCAATGGCACCTT	54.15	57	240	GA	4	130–170; 170–230	P	3
5	CNMK765	F: AATGTTATGCGAACCCTTGC R: TGACAAGGTGGACACTTGGA	55	53	211	GA	5	210–265	P	4
6	CNMK995	F: AAGACACGACACCCGATACC R: AAGTGCAACAGCACAACTGG	56.55	54	196	AG	6	190–242	P	2
7	CNMK1095	F: CCTCATCGGCTAACCTCAAG R: AGTCCTGAACCGAGGTACGA	56.25	54	229	CT	7	230–250	P	4
8	CNMK3414	F: CCAGCTCACTGCCATACAGA R: AGCACCAGGCTCTCATCTTC	56.85	50	289	AC	35	290–380	P	2
9	CNMK3683	F: ACCTCCCAGTGAAGACATGC R: CCGTTACGTTATCCCTTCCA	55.65	54	360	TG	40	320–370	P	2
10	CNMK4036	F: TGAGTTTCCTGGACCAAAGC R: ATACGCTGCTAGGGTGGTGT	56.8	55	254	GA	45	290–320	P	3
11	CNMK4153	F: CATTGGATGTGCCATAGTGC R: AGGTATGCCCAAGGAAAGGT	55.2	53	388	TC	46	380–400	P	2
12	CNMK4627	F: TGCGTGACCAACTACTCGAT R: GGAGCATGATGGGAGAGAAA	55.25	51	237	AG	56	220–245	P	4
13	CNMK4772	F: TGCATCAAGACAGACCATCC R: TGCATGAATACACCCACATT	53.5	59	351	GT	59	380–450	P	3
14	CNMK4830	F: AAATGGCTTTGCATGTCTCC R: GGTTGTCAAGCACCTTCCAT	54.75	53	341	AG	60	320–400	P	2
15	CNMK4976	F: TGCCACCATATGAGCAGAGA R: CCCAGAGCTCCAAACTCATC	55.65	58	199	AC	64	170–200; 215–250	P	6
16	CNMK5103	F: AACGTGTCCACTCTCCCAAG R: TTACCCGCCCTTATCTTCCT	56.15	54	307	CT	65	300–350	P	3
17	CNMK5211	F: AAGCTGACAAATGTGAAGAGC R: CAACAACGGTCTAGCACTGG	54.75	51	388	CT	68	190–210; 242–310	P	6
18	CNMK5746	F: CTGGGCAATGACACAACAAG R: AACCGACACAGAGAGGATGG	55.45	50	152	CT	80	380–400	P	4
19	CNMK5910	F: CGATTGAAGCTGATGCTCTG R: TTGATGTGCGTGAATGGAAT	53.25	53	338	AG	82	330	M	1
20	CNMK6063	F: CCACAACATGAAAGCAAGGA R: TGGACTAGGAGTGGGATTGG	54.7	53	394	AC	86	390–450	P	2
21	CNMK6206	F: TGCTAAAGGACCGGAAGATG R: TCATGGAGAGGTGCATGTGT	55.4	53	321	GT	89	300–340	M	2
22	CNMK6376	F: CATCTCCTAAGCTGGCAACC R: TAGGATAGGTGACGGCAACA	55.6	53	368	CT	92	380–450	P	3
23	CNMK6463	F: GATACAGGTGGCCAGGTGAG R: TCCAGCACTCTGTGCGTTAT	57	57	304	GA	96	230–240; 300–320	P	4
24	CNMK6507	F: CCCAATACCTTTGGTTGCTC R: TGCATGTCCGTGCATAAG	53.4	51	336	GA	98	150–160; 180–200	P	5
25	CNMK6571	F: TGAATGCCGTTGTTTGTACG R: GGATAAGGTCTGCATGGCTAA	54.15	62	398	CT	99	400–450	M	1
26	CNMK6672	F: ATATAGGCAAGGCCCAAGGT R: TGGTCATGGTGGAGTAGCAA	56.05	56	363	GA	102	330–370	P	4
27	CNMK7007	F: GGTGATTTCGTCGTCTCGTT R: TGCAGAATGCTTGGCAATAG	54.3	57	381	TG	109	350–380	P	2
28	CNMK7162	F: ACGCACACCTGAAAGGTACA R: GTTGCGAAGGATTTGCACTC	55.65	56	259	GT	113	250–320; 400–420	P	3
29	CNMK7449	F: TGCATCACACAGCTACACCA R: GTGCAGTTTGCTGGTCTTCA	56.3	54	246	TC	119	240–260	P	4
30	CNMK7553	F: TTGAGGTGTTCCAAACATGG R: GCTTGTAGGGCACGTTCATT	54.5	50	267	AG	121	240–260	P	3
31	CNMK7710	F: TCAACTGCATCAGGTCTTGG R: CTGACAGGTGGCACAGAGAA	56	54	301	GA	127	220–230	P	3
32	CNMK7859	F: ATGGTCTGGATATGGCTTGC R: TCTCCGTTCACTCTGCAACA	55.65	54	355	AG	132	300–310	P	2
33	CNMK8015	F: GGCAGTTCCACTTACCCAAG R: TGCTTAACAAAGCGTTCGTG	54.9	55	391	TG	135	250–320; 400–480	P	2
34	CNMK8444	F: GGTCCGGGATTCAGGTTAGT R: AGCCAAAGAACCCTTGGAAT	55.5	54	295	GT	152	200; 250–320	M	1
35	CNMK8741	F: ACCCGAGGTTTGAAAGGAAC R: TTGGCACCTTCACTTATTGC	54.35	57	248	TC	166	320	M	1
36	CNMK9091	F: GCTTGATTCCCTGGATACGA R: CTTGCTCTGTTCCCCATGAC	55.1	58	373	AG	185	320–380	P	2
37	CNMK9331	F: ATGCTTCGCTTGGTTGTAGG R: AGTGAGGAATCCGATGCAAT	54.8	45	374	GA	196	350–380	P	2
38	CNMK9514	F: TGAGATGAGATGGGTGGACA R: ATCAATGGGAGGTCACAAGG	54.95	50	324	TG	200	150; 220–240	P	3
39	CNMK9655	F: TTGGTCTAGTCCTGCCATAGG R: CCAATCAACACCCACATTGA	54.55	50	381	AG	207	242–280; 370–400	P	2
40	CNMK9918	F: TTGGACTCCCAACGACTAGG R: TTCCTTCCAAGCAGATGTCC	55.5	56	254	GT	224	230–242	P	2
41	CNMK10005	F: TGGTGCAGTCTTCTCAATCG R: CCATCTTCTCCCTGATTCCA	54.35	54	251	CT	229	242–260; 320–350	P	2
42	CNMK10146	F: AATCGAAATACGTGGCGAAC R: GCTTGTAGCAACTCCAACGTC	54.9	58	208	GA	237	210–225; 240–250	P	3
43	CNMK10298	F: TCACTCCATCACCCAAAGAA R: TTTAGTCCCAAGTGCCCATC	54.2	54	237	AG	245	230–245	P	2
44	CNMK10608	F: AGGAACTCATCGGTGTTTGG R: GCATGATTGTTGCATTGGAG	53.85	53	389	GA	265	350–380	P	2
45	CNMK10723	F: GTTTGCAGGTGGAAAGTCGT R: AGCTTCTTGATGCCATAGCC	55.55	56	324	TC	274	280–350	P	3
46	CNMK10821	F: AATACGCCACGTTACCCTTG R: TGCAGTGTGGAAGACACCAT	56	59	326	AG	280	310–350	P	3
47	CNMK11095	F: GATCGGCACTAGGGAACTTG R: AATGCGAGGACAACTGGAAC	55.4	55	361	AG	295	330–360	P	2
48	CNMK11349	F: ATGGCATTTGAGGATGAGAA R: ACCGTTCTTTGGGAAATGTG	52.55	62	280	AC	308	200; 300–350	P	3
49	CNMK16404	F: GGAATCTGAAGCAGGGACAG R: AGGGCATTGAAGAACAGCAC	55.6	54	334	AG	1122	300–400	P	3
50	CNMK16553	F: GCCGAAATTGTCTTAATAGGTG R: AGGAATGCCATGTCAGGTTC	53.45	48	339	GA	1168	200; 320–340	P	3
51	CNMK16634	F: CGAGCTTGAAGTCAGCTTTG R: GCAGCCTTACCTCTCACGTC	55.95	54	357	AG	1203	400–500	P	2
52	CNMK17050	F: ATTGGCTGAGTGGAGGACAA R: ATGAGCAACCCATGTTGATG	54.85	52	397	CT	1370	400–470	P	2
53	CNMK17101	F: CCAGCCATGCTTACCAACTTA R: CGAGAACCACGTCAATGAGA	54.9	50	299	AG	1401	250–290	P	3
54	CNMK17156	F: GTGTTCTGGCAATCATGCTC R: TGCTTGACATACGCACACAA	54.7	53	293	GA	1418	240–265	P	3
55	CNMK17229	F: CAAGCTGGAGACAACACAGG R: TGTCATCGACGAACTGGAGA	55.75	56	400	AC	1462	280–320	P	2
56	CNMK17487	F: TGCCAATGTGTTAAGGATGC R: TCCATACGAAGGCAACTGTG	54.25	52	288	CT	1550	310–350	P	3
57	CNMK17639	F: AAATCTGGGTGGCTCTCTCA R: GCCAGCAGCTATGGAAGAAG	55.85	54	303	TC	1621	280–340	P	4
58	CNMK17725	F: GCTGAGTTGCTTGTTGTCCA R: CCTGAACCAAGGGATGAGAA	55.1	55	234	AG	1655	170–190; 220–250	P	6
59	CNMK17797	F: GGTGCCTTAGTGCCTTCTCTT R: AATCCGTTGCGACGTTATTC	55.25	53	370	GA	1688	315–350	M	2
60	CNMK17875	F: GGCTTGGGGTTCAAATTCTT R: TTGAAGGCACCTAAGGCACT	55.1	50	397	AG	1750	300–400	P	2
61	CNMK18331	F: TGCAAAGTAAGGACCCTGGA R: CTGATTCATGGTTGGCTCCT	55.45	55	368	AG	1987	320–500	M	1
62	CNMK18501	F: CTGGACGAAACAATGGTCCT R: CTCCAGAGGGTATCCATCCA	55.25	55	380	TC	2084	350–400	P	2
63	CNMK18573	F: TGTGTTCGACTCGGTCGTTA R: AGGCCTTCTTCGATCACTAGA	55.45	57	383	TG	2130	400–440	P	2
64	CNMK18799	F: GCCTGCATTATTCACCTGGA R: GTCAGGAGGCAGTGGAGAAG	56.25	56	255	AG	2306	240–320	P	2
65	CNMK18903	F: CAGGACTCGGGAGATAGCTG R: TTGGCTGCTAATGTCTGCAC	56.2	56	396	AG	2370	350–400	P	2
66	CNMK18972	F: GGTGGTTGGCATCCTATGTT R: TACGTGGGGACACCAAGAGT	56.6	59	358	TC	2409	230–260; 330–400	P	5
67	CNMK19118	F: TACCCATCCCACAAATGGAC R: TCAGGGTGGCATGATGAATA	54.1	52	393	CT	2535	230–320	P	4
68	CNMK19193	F: ATGTTGTGGGGACGATGAGT R: ACCTCGCATGAGTGAAACTG	55.75	54	398	TC	2585	360–400; 690	P	3
69	CNMK19386	F: AAGGGTTTGAGTTGGTGGTG R: CCTAACCAGGCAAAGGACAA	55.25	52	354	AG	2748	300–330; 400–420	P	3
70	CNMK19611	F: ATCCATCCAATGCTATCAGG R: GACCGCATTAGCTCTGGTACT	54.2	50	364	AC	2872	300–340	P	3
71	CNMK19799	F: CGTCTGGGATAGCCTTCAGT R: CCAAGCAACGGAGAACTTG	55.2	55	337	TC	2988	320–410	P	3
72	CNMK20018	F: TGACAAGTTTCAGGGCATCA R: TGCAGATCTTGCCAAACGTA	54.5	53	362	AG	3251	300–330	P	3
73	CNMK20227	F: GCAGCACACTCATGCAAAAT R: TTAGTGGCGAGAGAGGTTGG	55.55	54	278	TG	3455	320–400	M	1
74	CNMK20739	F: ACACGATTGATGCATGAGGA R: GCCATGAGCCCACATCTATT	54.6	58	270	AG	4154	250–330	P	3
75	CNMK21015	F: CATGCCATTTGTCAATCCA R: TGCAGAGGAGTCCAGTGATG	54.05	57	333	CT	4606	130–160; 310–500	P	2
76	CNMK21174	F: CATGACTGACCGCTCTACTCC R: TCCTAATCCTCCATGTTGCTG	55.65	62	392	AG	4907	380–400	P	4
77	CNMK21493	F: AGGCATAGTCTCTCGGCTTG R: CAAGTGGAATTGCTCGTGAA	55	55	315	TC	5710	220–230; 320–400	P	2
78	CNMK318	F: GGCAAACCTTCCTAAATGACC R: CTTTGTCCAGCCGTACCTGT	55.6	60	358	TG	1	310–330	P	2
79	CNMK425	F: GGATGTAGGTTGGGCTCTTG R: GCCACTAGAGGGTCATTGGA	55.9	59	217	TG	2	130–160; 180–200	P	3
80	CNMK808	F: CCATGCCACACCTTACTCCT R: AACAAACGCCCACCTATCTG	56.1	55	225	GA	5	280–320; 400–430	P	3
81	CNMK3765	F: GAGAGAGTGGTCGGCTTCAC R: ATTCGGATGTTCGATTTGGA	54.7	55	372	TC	40	290–380	P	3
82	CNMK4127	F: TCAACGCATCAATACCCAAG R: GAAGTCCAAGCAACCAGCTC	54.65	58	274	TG	46	160; 350–390	M	1
83	CNMK5054	F: CTTCATGGTTCATGGTGCAG R: ATGGATATGAACAGCGAGCA	54.1	57	391	TG	64	400–440	P	2
84	CNMK5329	F: GCTGGTCGGAAATGCTAGAC R: CATGCAAAGCCTCACTCAAG	55.05	53	320	ACAT	70	300–320; 360–410	P	2
85	CNMK5632	F: TAGCCCTTTCAGGACCCTCT R: GAGGAAGTCATCCGAAGCAG	56.4	56	180	TG	78	180–200	P	5
86	CNMK6746	F: AAGCACTTCCTTACGCCAGA R: GCCTTGGTGGTGAAGATTGT	56.05	59	382	TG	103	380–400	P	2
87	CNMK6908	F: AGATTGCCGGAGTTGATTTG R: CCTATTCGGTCGCAATTGAT	53.05	58	344	CT	107	200–300	P	3
88	CNMK7627	F: TTGAGTCTGGAATCGTTAGAGG R: CATGGTGGCGAACTGTGATA	54.45	54	356	TG	124	240–350	M	1
89	CNMK7985	F: GAAATGAGACCGCCATTGAT R: CGGACCGTTAGACAGATTGC	54.4	57	323	AC	135	320–340	P	2
90	CNMK8083	F: GGCGTATTCGGTAGCATCTC R: CTCCAGCACAGATGGAAACA	55.15	58	247	GA	137	200–250	P	3
91	CNMK8371	F: AAGGACTTGTGATGCCTTGG R: GTCACCATAGCCGACAACCT	56.2	54	310	AG	148	310–400	P	3
92	CNMK8904	F: GTTTGCCCGTACTTCTCAGC R: TGGCAGCATCACTCTTTCAC	55.85	56	364	GA	178	320–350	P	2
93	CNMK9440	F: TGGGACCTGTCCTGCATATT R: TATCGGCACATTCGATTTCA	53.9	54	333	GA	199	300–340	P	2
94	CNMK9988	F: CTCGCAAATGCAATATAGGC R: CGCAAATTCGGTTGATCTTA	51.45	49	383	GA	228	230–320	P	4
95	CNMK10263	F: TTCAGGACAATTGGAAGTGTTG R: AGAATGCCCAAGTCAAGCAG	54.5	55	313	CT	244	290–320; 400–500	P	5
96	CNMK10632	F: TTCACGTTGCCAAATGACAC R: CAGGCATGCACTCAAAGATG	54.25	52	376	AC	268	150	M	1
97	CNMK10681	F: CGACCTCCATACATGGCTCT R: TAACTGGCTTTGGGTTGGTC	55.85	57	337	TG	269	320–400	P	3
98	CNMK11807	F: AGTGAAGATCTGCCCGAGAA R: TTTGAACTCACGCTTGTTGC	55	55	305	CT	346	242–330	P	3
99	CNMK12241	F: AGTGCTAGCCAGACCCATGT R: CTCCCAAAGGTACGTGCAAT	56.9	55	315	AG	385	240–330	P	4
100	CNMK12746	F: TAGATCGAGGCATGCGAGTA R: AGAATGGATATTGGCCCTCA	54.3	54	383	TG	441	380–450	P	2
101	CNMK13043	F: CCTTCAGGGTTAGGTGCAGA R: TCAAACTGGCTGATCCTTCA	55.25	55	383	AG	472	320–340	P	3
102	CNMK13232	F: GGAAGTCCTCAGTCGTGCTC R: GGAAATCAAGAAGGCATTGG	54.7	58	165	TC	495	170–230	P	3
103	CNMK13632	F: CGAGGGTCTCAGCGATTTAG R: GCTGGACCTTTGTGGTGAAG	57.2	57	379	TG	561	300–330	P	3
104	CNMK13852	F: TTGGAAGAAATGGCAGTGGT R: GGATATGGATGGATGGATGG	53.55	52	184	AG	595	390–450	M	2
105	CNMK13946	F: GGCAGAGGTAGTGGAACGAG R: CCTGACGGAGGACTGTTGAT	56.9	57	363	TC	605	330–400	P	3
106	CNMK14272	F: AAGGGTGCATGATGGTTAGG R: CAAACATTCCTCCGTGTGTG	54.55	53	371	AG	643	320–410	P	2
107	CNMK14692	F: GGAGGCTACCAGCCATAGTG R: CACAGTCCTCTGCGATGAGA	56.85	53	370	GA	700	330–380	P	2
108	CNMK15137	F: TTGGTCGCATGATTGTCTTC R: CTGAGCACCCTGTGGTAGGT	56.1	56	337	TC	796	330	M	1
109	CNMK15508	F: ATAGCTGGTGAGTCGGCAAG R: GGCCTACTGATTGGACTGGA	56.75	57	374	AG	868	200–220; 320–400	P	3
110	CNMK15694	F: AAGACTGTTGCCCTGGAAGA R: TCGATGATGCAGAGATCAGG	55.15	55	353	AG	908	320–390	P	3
111	CNMK15970	F: CGTGTTGGTGATTGTTGCAT R: ATTGCGGGGTAAGGAGAAGT	55.15	53	294	TG	994	220–280	P	3
112	CNMK5852	F: ACCCACTAGCACTTGCACCT R: CCTGAGGTCAACAAGCCATC	57.4	60	310	AG	82	310–400	P	3
113	CNMK17532	F: AAGTTCGGCTCACCAATCAC R: GATGGGGATACATCCAATGC	54.4	55	388	AG	1570	350–500	P	2
114	CNMK17684	F: TAGCCGTCCGATATTCAAGG R: TGCATTCTAAGGGAATGGATG	53.3	53	176	CT	1636	170	P	2
115	CNMK18364	F: TCCCAATGGCAGTCCTAGTC R: GAAACCCATCCTTGTGGAGA	55.45	58	347	GA	2021	260–320; 470	P	4
116	CNMK19333	F: ACCTGCCTATTCATGCACAA R: GAGTGACGCAAGACAATCTCC	55.15	53	304	AC	2726	280–420	P	4
117	CNMK19929	F: AACTGAGCAGATGGGCTTTG R: AGCCTCTGTGACGAACGAAT	56	56	321	AG	3153	260–330	P	3
118	CNMK20075	F: GTTGTGCCTCCAATGTTCCT R: GATCGGCCTGAATCCTGTAA	54.95	55	280	GA	3333	300–320	P	4
119	CNMK1433	F: GGTGATTGACTCCTGGCACT R: TTCACCTCTGGATTCTTGGTG	55.85	50	183	GA	10	150–170	M	1
120	CNMK1524	F: GCTCCTGGTACAGGCACATA R: ACATCGGGATGGGTTCAAT	55.3	55	266	GA	11	230–250	M	1
121	CNMK1644	F: TAGGACGTTTACCGCAGGAG R: CTGTAGGGTAGGGTGCATCG	56.8	57	205	TC	12	190–210; 270	P	4
122	CNMK1809	F: CGGGACTTGGGAGTCATCTA R: TGGCACTTCGTCTGTGTAGC	56.6	57	386	AG	14	350–500	M	1
123	CNMK2363	F: CAAGACACAGCTTCGAGATCC R: GATTCCTCCGCCTATGACCT	55.85	56	400	AG	22	350–380	M	1
124	CNMK2470	F: GCAATCGAGCCCAGAACTAC R: CCCAACCTTCCACCAATATG	54.6	55	369	CT	23	250–300	M	1
125	CNMK2651	F: CCTCCCTTCACCTTGCATAA R: CTGCACTGCTCACCGTATGT	56.05	54	306	GA	25	280–320	P	3
126	CNMK2960	F: GAGGAGTGAGACGGATTGGA R: CGATCCAAGATTGGTACTGGA	55.05	55	300	CT	30	260–300	P	3
127	CNMK3730	F: CGATTGAAGCCCAGTCTCTC R: AACGACATCTTCACCAGCAA	54.95	53	296	AAG	40	250–280	P	3
128	CNMK3865	F: AGGAGTAGCTCCGCCCTCTA R: CCCTCGAATGACCAGAGAAG	57.05	57	323	AG	43	300–320	P	2
129	CNMK4080	F: AGTTTCGTAGCGGCTGATGT R: AGAGCACTCAGCAAGCAGGT	57.9	53	327	AG	45	300–320	P	4
130	CNMK4336	F: CCGACGTGTTGACAGCTCTA R: AAACCTTTCGCACGAATCAC	55.35	55	229	AC	49	230–300	P	4
131	CNMK5287	F: CCCAACAGACCCAACTCAAT R: TGTGGAAGATGTGGAGTGGA	55.4	53	205	CT	69	230–340	P	4
			N/A	N/A	N/A	N/A	N/A	N/A	N/A	2.7

*M *monomorphic, *P *polymrphic, *N*/*A *not applicable

^a^Based on gradient PCR optimization

## Discussion

The work of Lantican et al. [9] was able to identify genome-wide SSRs based on de novo prediction of repeat loci across the CATD genome assembly. However, the predicted loci were not screened nor tested in actual wet lab conditions. Here, the SSR markers generated were subjected to various filtering parameters that are advantageous based on genome distribution, repeat motif, and ideal thermodynamic properties. Markers with AT/AT and TA/TA repeat motifs were excluded in the selection since these are the most common type of repeats found in the coconut/palm genome [9, 13, 29] on which the high repeat content may hinder specificity of the markers and/or may result to nonspecific amplification of products. Markers were also selected based on the distribution in the contig to cover the entire coconut genome. In silico PCR in the CATD genome assembly was performed. This allows checking of contig specificity of the marker and ensures in vitro SSR amplification [22]. Allele size range of the markers was also limited to 80–400 bp for easy visualization in gel, and OligoAnalyzer tool was used to check dimerization capability and formation of hairpin loop of the primers to produce high-quality markers.

The predominance of dinucleotide repeats in coconut and other related species is supported by previous works of Rivera et al. [21], Palliyarakkal et al. [13], Xia et al. [29], and Lantican et al. [9]. This result coincides with studies of Palliyarakkal et al. [13] and Xia et al. [29] on which AG/GA/TC/CT motifs were also the most common dinucleotide repeats found in coconut/palm genome. The results obtained here are consistent with previous studies on which high levels of polymorphism are likely attributed to phenotypic variation and differences in the breeding behaviors of the dwarf and tall varieties which are said to be generally autogamous (self-pollinating) and allogamous (cross-pollinating), respectively [14, 21, 25]. The development of SSRs using advanced bioinformatics tools in this study has become very efficient in generating high number of markers in coconut. The generated SSRs here are expected to contribute to the pool of available molecular markers [10, 16, 28–30] for fingerprinting, genetic diversity analysis and QTL mapping, and other relevant studies in coconut.

Microsatellites or SSRs are a very useful molecular tool for studying genetic diversity and genotyping of coconut [8, 10, 15, 16, 30]. It has been extensively used in these analyses since SSR markers are abundant and well distributed throughout the genome, multi-allelic, co-dominant, highly polymorphic, and highly reproducible [11, 20]. Previous studies like Rivera et al. [21], Perera et al. [15], Xiao et al. [30], and Wu et al. [28] have already developed SSRs in coconut for genetic diversity studies, and these markers showed high levels of polymorphism as well.

## Conclusion

Here, we demonstrated that a locally established bioinformatics pipeline can mine SSRs from NGS data with actual utility in terms of amplification and distinguishing power across several varieties of coconut. The advantage of using a genome-wide bioinformatics prediction approach in marker development is its relatively fast and cost-effective way of generating vast amounts of markers. SSRs and SNPs can be easily generated automatically in the genome sequences with the use of these programs or pipelines.

Polymorphic markers in this study will be further used to genotype the coconut mapping population generated from a three-way cross of ‘Pacific’ LAGT and CATD and ‘Indo-Atlantic’ WAT coconut for QTL mapping analysis. The development of novel SSR markers for coconut will serve as a valuable resource for mapping QTLs, assessment of genetic diversity and population structure, hybridity testing, and other marker-assisted plant breeding applications.

## Data Availability

The dataset(s) supporting the conclusions of this article is (are) included within the article (and its additional file(s)).
